# The Evolving View of Coronary Artery Calcium: A Personalized Shared Decision-Making Tool in Primary Prevention

**DOI:** 10.1155/2019/7059806

**Published:** 2019-06-02

**Authors:** Omar Dzaye, Cara Reiter-Brennan, Albert D. Osei, Olusola A. Orimoloye, S. M. Iftekhar Uddin, Mohammadhassan Mirbolouk, Michael J. Blaha

**Affiliations:** ^1^Johns Hopkins Ciccarone Center for Prevention of Heart Disease, Baltimore, MD, USA; ^2^Russell H. Morgan Department of Radiology and Radiological Science, Johns Hopkins University School of Medicine, Baltimore, MD, USA; ^3^Department of Radiology and Neuroradiology, Charité, Berlin, Germany

## Abstract

The 2018 American Heart Association and American College of Cardiology (AHA/ACC) cholesterol management guideline considers current evidence on coronary artery calcium (CAC) testing while incorporating learnings from previous guidelines. More than any previous guideline update, this set encourages CAC testing to facilitate shared decision making and to individualize treatment plans. An important novelty is further separation of risk groups. Specifically, the current prevention guideline recommends CAC testing for primary atherosclerotic cardiovascular disease (ASCVD) prevention among asymptomatic patients in borderline and intermediate risk groups (5–7.5% and 7.5–20% 10-year ASCVD risk). This additional sub-classification reflects the uncertainty of treatment strategies for patients broadly considered to be “intermediate risk,” as treatment recommendations for high and low risk groups are well established. The 2018 guidelines, for the first time, clearly recognize the significance of a CAC score of zero, where intensive statin therapy is likely not beneficial and not routinely recommended in selected patients. Lifestyle modification should be the focus in patients with CAC = 0. In this article, we review the recent AHA/ACC cholesterol management guideline and contextualize the transition of CAC testing to a guideline-endorsed decision aid for borderline-to-intermediate risk patients who seek more definitive risk assessment as part of a clinician-patient discussion. CAC testing can reduce low-value treatment and focus primary prevention therapy on those most likely to benefit.

## 1. Introduction

The aim of this article is to review the 2018 American Heart Association and American College of Cardiology (AHA/ACC) cholesterol management guideline, and to place new recommendations on coronary artery calcium (CAC) in greater context. The role of CAC for cardiovascular risk prediction differs from the 2013 to the 2018 AHA/ACC guidelines and reflects the considerable amount of research development on CAC scoring over the last years [1]. Estimation of atherosclerotic cardiovascular disease (ASCVD) risk using the Pooled Cohort Equations (PCE) remains an important step in clinical decision making for primary prevention of ASCVD in asymptomatic individuals [2]. Additionally, it provides an avenue to identify patients who might benefit from preventive pharmacotherapies. Although the 2018 AHA/ACC cholesterol management guideline recommends the use of these equations, it acknowledges its limitations with risk discrimination and overestimation. Studies have shown that these equations exhibits just moderate risk discrimination, and commonly overestimates ASCVD risk [3]. This may be especially true among non-Caucasian and non-African American populations, and also among older populations since PCE is heavily weighted towards patient age [4]. However, AHA/ACC guidelines assigned a class IIA recommendation for the use of supplementary tools, such as CAC scoring, for more accurate risk assessment beyond the PCE “when risk or the decision to treat is uncertain” [5–8]. Head-to-head comparison of CAC with other traditional risk factors in the Multi-Ethnic Study of Atherosclerosis (MESA) has shown that CAC is the best prognosticator of coronary heart disease (CHD) risk [1, 9, 10]. The Dallas Heart Study (DHS) has observed similar results [11]. In the Heinz Nixdorf RECALL (HNR) study it has been shown that persons with severe CAC had higher hazard ratios than those with a CAC score of 0 [1, 12]. CAC scoring has been demonstrated to be useful for re-classifying risk and moving patients to lower or higher risk groups.

More endeavors have been required to understand how to convey CVD risk estimation and to use these approaches for shared decision making as current approaches for the prevention of ASCVD are explicitly risk-based [1, 7]. So far, traditional risk factors were incorporated in cardiovascular risk assessment (e.g. gender, family history, smoking status, age, diabetes, total cholesterol and HDL). However, risk calculators that exclusively include traditional risk factors have moderate risk calibration, and commonly overestimates ASCVD risk. In the United States, risk estimation begins with the PCE, which were first introduced in the 2013 AHA/ACC guideline [6]. The new 2018 AHA/ACC guideline still recommends use of these equations as a prudent first step in clinical decision making, despite acknowledging that they provide only moderate risk discrimination [4, 8].

CAC has nowadays extended from traditional risk prediction studies to patient-centered research with direct implications for personalized clinical practice. CAC scoring measures ASCVD risk by capturing lifetime accumulated exposure to measurable and unmeasurable risk factors. Therefore, it is a strong surrogate of total burden of atherosclerosis. The ability to effectively detect CAC resulted in a paradigm shift in cardiovascular risk calculation, as it allowed direct measurement of subclinical disease, instead of only paying attention to one time measurement of traditional risk factors that only partially reflect an individual's true risk [13]. Since publication of the 2013 guideline, concern has been raised that risk overestimation could lead to statins being recommended to many patients who are less likely to receive net benefit from therapy. For patients at either high or very low risk for ASCVD, imprecise risk estimation may not be clinically relevant. However, for all other patients, using the PCE as a standalone risk assessment tool may be insufficient for definitive decision making [2].

This central limitation is echoed in the current AHA/ACC prevention guidelines of 2018, as these specifically state that “identification of subclinical atherosclerosis rather than use of serum biomarkers is preferred, because of the extensive body of evidence demonstrating the superior utility of atherosclerosis disease assessment” [8]. Consideration of additional clinical factors and other tests to more accurately assess cardiovascular risk for many adults with 10 year risk for ASCVD between 5% and 20% are recommended. The presence of these risk enhancers, such as rheumatologic disease, HIV infection, South Asian ancestry, inflammatory biomarkers and a family history of premature ASCVD, can increase risk. However, risk enhancers are only valuable for identifying persons who may be at higher risk than otherwise expected. Their absence does not reclassify risk downward, and borderline to intermediate risk patients especially those with no traditional risk factors may still face risk overestimation and consequently potential overtreatment. [2, 8, 14].

## 2. Current 2018 AHA/ACC Cholesterol Management Guideline

The current 2018 AHA/ACC cholesterol management guideline implements learnings from previous guidelines and current evidence on CAC testing. More than any previous guidelines, this set encourages CAC testing to implement shared decision making and to individualize treatment plans [15]. Specifically, the current prevention guideline recommends CAC testing for primary ASCVD prevention in asymptomatic patients and in borderline and intermediate risk patients (10-year ASCVD risk 5–20%). Lifestyle modification should be the focus in patients with CAC = 0. Statin therapy is recommended for patients with a CAC score between 1 and 99 and strongly indicated at a CAC score >100 or if patients are in the >75th percentile. The guidelines suggest that CAC testing can be repeated after 5 years if the CAC score is 0 or 1–99 [8]. An important novelty is further separation of risk groups. The 2018 AHA/ACC cholesterol management guideline consider individuals with a 5% 10-year ASCVD risk as low risk, 5–7.5% as borderline and 7.5–20% as intermediate and >20% as high risk. This additional sub-classification reflects the uncertainty of treatment strategies for patients with intermediate risk groups, as treatment recommendations for high and low risk groups are well established [15]. As suggested by earlier guidelines, the 2018 AHA/ACC cholesterol management guideline emphasize that “clinical judgment and patient preferences should guide decision making.” Importantly, the new guidelines recognize that the CAC can be used to increase as well as decrease risk scores of patients, while the CAC in previous guidelines was used to select high risk patients for more aggressive treatment. Multiple studies have suggested the effectiveness of CAC testing for both upwards and downwards reclassification of ASCVD risk. The net reclassification index (NRI) was 0.66 for CAC in intermediate risk patients, while it was 0.02–0.1 for other biomarkers [16]. More studies have shown that in a group of individuals with 10-year risk of 5–20%, 50% can be reclassified with CAC testing [17, 18].

The 2018 guidelines, for the first time, more clearly recognize the significance of a CAC score of 0, where intensive statin therapy is not beneficial and not recommended. This update is a response to multiple studies demonstrating the high negative predictor value of CAC = 0, also known as “the power of zero.” For instance, in a study evaluating 13 risk factors using data of the MESA study, CAC = 0 was the strongest negative risk factor [19]. Moreover, a CAC score of 0 was found to be the greatest factor of downward risk reclassification among all negative risk parameters like low levels of high sensitivity c-reactive protein or low ankle brachial index [20]. The current guidelines also state that CAC testing for further risk stratification is not suitable for diabetics, smokers, patients with premature cardiovascular disease. The 2018 guidelines emphasize that CAC is not a screening tool but an extra tool which helps to “minimizes” patients in risk, and discriminates individuals, who do not profit from intensive statin therapy regime [2].

## 3. Conclusion

In summary, CAC scoring has evolved from a research tool to a firm part of the decision algorithm to create individualized therapies. It is the most valuable test to reduce low value treatment and offer primary preventive care to patients who will truly benefit. The development of the role CAC over time can be observed through guidelines changing recommendation on CAC testing. Originally, guidelines recommended CAC as a tool to identify high risk patients for additional risk stratification [15]. Current guidelines however recommend CAC testing for ASCVD (5–20%) intermediate risk patients. CAC is recommended by present guidelines to implement shared decision making on order to design optimal treatment plans for each individual patient. Health care professionals should ensure that patients are informed about all available options in the context of a risk-based approach. The shared decision-making process means not only to include sharing the best scientific evidence with patients but also considers patients values and preferences. Therefore, CAC scoring is an option that should be made available for intermediate risk patients who desire additional risk information. Safety concerns regarding CAC scoring, such as implications of potential incidental pulmonary findings or radiation exposure, vs. benefits of more accurate risk stratification to start lifelong statin therapy, should be part of the shared decision-making approach. Shared decision-making provides patients with the opportunity to weigh pros and cons of treatment without or with further testing and improves potentially their engagement in disease management [1].

### 3.1. Take-Home Message

**Figure 1:**
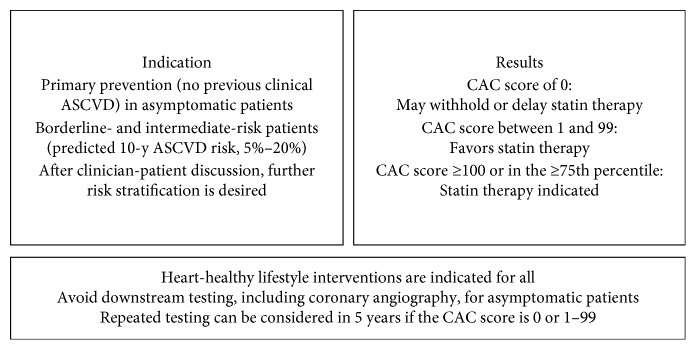
Coronary artery calcium testing.

It is critical that physicians understand the newly proposed role for CAC testing and do not equate it with screening. Rather than bringing in many additional statin candidates, this testing should serve as a decision aid to “de-risk” certain patients and distinguish those who may benefit from preventive pharmacologic therapies. The updated 2018 AHA/ACC cholesterol management guideline strongly endorses selective CAC testing, but the decision to use this testing is not always straightforward. CAC testing is now a guideline-endorsed decision aid for borderline-to intermediate risk patients who seek more definitive risk assessment as part of a clinician—patient discussion. This testing can reduce low-value treatment and focus primary prevention therapy on those most likely to benefit (Figure 1).

## Abbreviations

ASCVD: Atherosclerotic cardiovascular disease
CAC: Coronary artery calcium
CVD: Cardiovascular diseases
DHS: Dallas heart study
HNR: Heinz nixdorf recall study
MESA: Multi ethnic-study of atherosclerosis
NRI: Net reclassification index
PCE: Pooled cohort equations.
